# Monthly Summary

**Published:** 1880-08

**Authors:** 


### MONTHLY SUMMARY.

*Recent Dental Conventions.*—The activity among the dentists has been unusually great during the past few weeks. The American Dental Association held an annual meeting at Boston, August 4th, 5th, and 6th; the American Dental Convention and the Southern Dental Association met in this city, August 10th to 14th. These two latter associations united to form a National Association under the name of the Dental Association of the United States. This association is expected to be the representative body of the American dentists. It will hold annual meetings in different cities, and quadrennial meetings in Washington, where its permanent secretary is to reside. The Association aims at high scientific work, and will arrange at once to secure governmental recognition to the dental profession, and help from it in studying ethnological peculiarities in their relation to dentistry. The Smithsonian Institution promises aid in this direction.

A vote was passed making it a requisite for membership that the applicant should have credentials from a State society, or the diploma of a reputable dental college. This latter regulation, it is expected, will give an impulse to dental education.

The new association was not formed without some opposition. The American Dental Association has tried to have a national character, and it refused to allow its organization to be absorbed in any other. But its work and membership, if we may believe the reports, have been of a too local character. It was therefore decided to form an entirely new society. Any genuinely scientific work done by the dentists will be of value to the medical profession. If this new association, therefore, secures, as it promises, the performance of such work, its creation will be a matter of congratulation for us.

We have examined the reports of the various meetings above referred to, and confess to being greatly disappointed in our hopes of finding any good scientific work performed. In the American Dental Association there were papers devoted to the relative merits of cohesive and non-cohesive gold, the respective values of which, it seems, ought to have been settled long ago; the use of belladonna to lessen the flow of saliva, catarrh in its relations to dentistry, and the evil effects of the various patent amalgams, were some of the topics presented. We noticed a paper on Anæsthesia, which was defined to be a "paralyzation of the nervous tissue." In a subsequent discussion on the subject, the question as to whether nitrous oxide oxidized the blood, or prevented its oxidization was discussed. The fact was brought out very strongly that the Association did not know anything about the matter whatever. A practical movement was made in the appointment of a committee to arrange for an International Dental Congress. Considerable time was devoted by the American Dental Association to the discussion of dental education. Of course the question whether dentistry was a branch of medical science was fully debated. We quote and commend the remarks of Dr. Barrett on this point: "We dentists," he said, "should be honest. We are not medical men, and are not acknowledged as such by physicians. There is no way by which a student can become a member of the medical profession, except through a medical college." The Association urged that the dental colleges require a higher preliminary medical education and two courses of instructions.

We find even less evidence of work at the New York conventions than at that held in Boston. The daily papers perhaps gave undue prominence to certain parliamentary infelicities, but these were certainly much too frequent for the credit of the societies.

Most of the time was given to excursions and the business of organizing the association. An interesting paper was read before the Southern Dental Association, on Mercurial Poisoning Caused by Sucking the Amalgam of a Mirror. Not much else of general scientific value, however, was presented.

It will probably strike any one who reads the reports of the dental conventions to which we have been referring, that American dentists are persons sadly deficient, as yet, in scientific as well as in parliamentary knowledge. Their meetings have, however, shown a commendable appreciation of deficiencies and desire for progress. And considerable work was done which will eventually secure scientific and educational advancement in dentistry.—*Medical Record.*

---

*Influence of Tobacco Smoking Upon Health.*—In the *Revue d' Hygiene* for November, 15th, 1879, Dr. Decaisne observes that the excessive use of tobacco causes in some subjects intermission of the beats of the heart and radial artery. Out of eighty-eight smokers who came under his observation during a period of three years, he found twenty-one cases of intermittent pulse, without any organic lesion of the heart. He states—

1st.—That none of the subjects who came under his observation had organic disease of the heart.

2d.—That none of them were in a state of health favorable to the development of intermittence in the beats of the heart.

3d.—That in nine cases the complete abstention from smoking tobacco was sufficient to restore the cardiac rhythm and the system to their normal condition.

He therefore says that the abuse of tobacco produces in some subjects a condition which he terms *nicotism* of the heart, which is manifested by intermissions in the beats of that organ, and in the pulsation of the radial artery. It is sufficient in some cases to suppress or diminish the use of tobacco in order to stop or diminish the irregularity in the heart's action.

Dr. Delaunay, in commenting upon Dr. Decaisne's paper in the *Revue d' Hygiene* for January 15th, 1880, calls attention to the influence of the emanations from tobacco upon pregnancy. In a poor district of Paris there is a manufactory of tobacco in which 2000 women are employed. According to the evidence of a midwife who has attended a great number of women employed in this factory during their confinements, they are peculiarly liable to miscarriages, which they attribute to the influence of the exhalations from the tobacco. Some of the women, who are not altogether dependent upon their earnings from the factory, leave it when they become pregnant until after their confinement. One woman who had twice miscarried while working at the factory, left it when she was in the fifth month of her third pregnancy. Her child was born alive at the proper time, but died soon after its birth. The mother did not return to the tobacco factory, and her fourth pregnancy terminated in the birth of a healthy child, who survived. During her first three pregnancies she suffered much from obstinate vomiting—due, perhaps, to the action of the tobacco.

From these and similiar data Dr. Delaunay deduces the following conclusions:—

1st.—Tobacco has a pernicious influence upon the health of children and mothers.

2d.—It impairs the health of pregnant women, and causes miscarriage.

3d.—It has the same noxious effect upon children weak from their birth.

4th.—It diminishes the quantity of milk and alters its quality for the worse, and, consequently, prevents the proper growth of the child, who, indeed, often dies, a victim to his mother's occupation.—*Medical and Surgical Reporter.*

*Anæsthesia in Dentistry.*—The New Jersey State Dental Association, at its recent meeting passed a resolution to the effect that its members should avoid the use of anæsthetics as much as possible, and that in every case requiring anæsthesia the greatest care should be taken to insure the safety of the patient by a preliminary examination into his physical condition. Under ordinary circumstances the precautions advised would be considered altogether unneccessary; but, coming from a body of dentists. and with the formal sanction of a deliberate society vote, they have more than an ordinary significance.

It need not be stated that dentists, as a class, are addicted to the free use of anæsthesia; neither is it necessary to assert that a large proportion of fatal cases, especially with chloroform, have been associated with dental operations. Hence, it appears peculiarly appropriate for a society of dentists to give the matter such serious attention as is indicated by the resolution to which we have referred.

The injunction to abstain from the use of anæsthetics whenever such a thing is possible will not have, practically, much force with dentists. The desire of a patient to avoid pain, and the inclination of the operator to oblige him, are two powerful elements which will always argue in favor of anæsthesia. Besides, the public are educated to the belief that for tooth-extraction pain is not necessary. The administration of some sort of anæsthetic is now considered to be a necessary condition of the operation, and the dentists who fail to meet the want are in danger of losing custom. It can hardly be expected that any dental operator, under such circumstances, will strain a point to persuade a patient not to be rendered insensible to pain; but, if the other part of the resolution is conscientiously carried out, viz., the preliminary examination into the physical condition of the patient, many fatal accidents may be prevented. —*Medical Record.*

---

*Compressed Mother-of-Pearl.*—M. Duvochel has invented a compressed kind of nacre or pearl made of the pulverized shell of the Halotis, solidified with gelatine. Thus prepared it will serve for inlaying or mounting in cabinet work, cartonnage, tabletture, and other industries, and the manufacture of fans, buttons, etc. This product can be figured, stamped, moulded by pressure, poured out in the liquid state, and, in fact, take all forms. It can be dyed in any color, polished and varnished by the processes used for tortoise shell, mother-of-pearl, and other analogous substances. To render the shells thin and friable, they are submitted to a strong heat, which separates them into thin scales; these are then pressed in the cylinders of a flattening roller, and afterwards pounded in a mortar. It is then sifted to get rid of the dust, and the powder is treated with gelatine, and shaped into any form required.—*Am. Jour. Indust.*

*Ready Way to Test Chloroform.*—The purity of chloroform for anæsthetic purposes being very important, the following simple tests are recommended by Dr. J. Regnauld: If chloroform be dropped upon paper and allowed to evaporate, the last portion, on being inhaled, has a characteristic pleasant smell, and leaves the paper perfectly dry. Impure chloroform has a disagreeable, irritating odor, which it imparts to the paper. Pure chloroform does not redden blue litmus, or give even a cloudiness with silver nitrate; but if the sample should do either, it contains hydro-chloric acid or the products of the decomposition of some other chlorides. Pure chloroform remains perfectly colorless when boiled with potash; the presence of aldehyde causes a brown coloration. When chloroform is shaken with concentrated sulphuric acid and allowed to stand for half an hour, the two liquids should separate into two colorless layers; the presence of alcoholic chlorides produces a brown coloration. The purity of chloroform may be judged by its constant boiling point, 60 8° Centigrade; the impure sample may boil above or below, according to the impurities it contains. The assigned specific gravity affords no certain test. Hoffmann's violet will dissolve completely in pure chloroform, but if there is a trace of alcohol the solution is of a beautiful purple color.—*Druggists Circular.*

---

*The Tasting Power of the Tongue.*—The tasting power of the tongue is not regularly distributed over all parts of that organ. According to the unanimous judgment of physiologists, the back part of the tongue is best qualified for this function, while there is a difference of opinion as to the tip of the tongue. The old observers have repeatedly said that a tasting power in the tip is limited to certain persons, whereas more recent ones affirm its presence in all men. In experimenting on the so-called "reaction time," Harr Vintschgau lately met with a case of limited tasting power in the tongue-tip, and this led him to a thorough investigation of the subject. The observations were made with solutions of chloride of sodium, sugar, quinine, and citric acid. The results were as follows: There are persons who are capable of accurately distinguishing all principal tastes with the tip of the tongue alone; others perceive with certainty the qualities of sweetness, saltness, and acidity, but less distinctly bitterness; others again can only with great difficulty distinguish tastes with the tip of the tongue; and finally there are individuals who cannot do this in the least.—*Druggists Circular.*

---

*A New Anæsthetic.*—The French journals contain an account of the successful employment of Prof. Bert's new anæsthetic, which consists of nitrous oxide 85 parts, and oxygen 15 parts, under tension. Prof. Labbe, the distinguished surgeon of Lariboisiere, having occasion to remove an ingrowing toe-nail, carried his patient to the "Aerotherapic Establishment" of Dr. Dauplev, and administered the anæsthetic in a chamber, the atmosphere of which had been compressed to 92 centimetres. After the lapse of a few seconds the patient became perfectly insensible, and the operation was performed and the dressing completed without the slightest manifestation of pain at the time or the development of any unusual symptom afterwards. Under ordinary conditions this mixture produces no effect upon the economy, while its employment under tension results in the speedy development of an anæsthesia which is profound enough to render surgical operations painless, and of so innocent a character as to preclude the possibility of a dangerous complication.—*Dr. Edward Warren(Bey,) in N. Car. Med. Jour.*

---

*Remedy in Muguet.* (*Thrush.*)—Dr. Vivier observes, in *Progres Med.*, March 27, 1880, that by far the best application in this disease—that of Bazin—has almost fallen into oblivion, which he attributes to the fadt that Bazin never published a formula. After numerous trials Dr. Vivier recommends the following, which constitutes a mode of treatment incomparably superior to any other: Distilled water, 25 grams; alcohol, 5 grams; and corrosive sublimate 60 centigrams. The patches of muguet are to be swept over by means of a pencil moistened in this, once, twice, or three times a day, according to the rapidity of the reproduction of the cryptogam. In spite of the poisonous character of the agent its application is made without danger and always with rapid success, unless the patches are inaccessible by being placed in the phraynx or at the back of the velum. The application has only one inconvenience—that it must be made by the practitioner himself; but the rapidity of the cure amply compensates for this.—*Med. and Surg. Rep.*

---

*The Action of Collodion on Temperature.*—We learn from the *Bull. Generale de Therap.*, June 15th, 1880, that Dr. Raducan has made some interesting observations on the effect produced on the general temperature by the external application of collodion; these he publishes in his inaugural thesis (*These de Paris*, 1879.) In health the effect on the central temperature varies with the site of the application. If the collodion be spread on either of the lower limbs the temperature is unaffected. But if the collodion be applied in such a way as to cover all the cutaneous surface corresponding either to the peritoneum or the pleuræ, an immediate and notable lowering of the central temperature is observed. Dr. Raducan thinks that the difference noted explains the therapeutic action of collodion in inflammation.—*Medical and Surgical Reporter.*

*Safety of Hydrobromic Ether.*—The new anæsthetic has not yet defined its comparative safety and merits. Its action appears to be more nearly allied to that of chloroform than of sulphuric ether. In the *Cincinnati Lancet* is reported a case happening in Philadelphia under the charge of Dr. Tevis, the subject being a stout, muscular man who inhaled four drachms and then after violent struggling yielded to its influence and suddenly ceased to breathe. Artificial respiration was at once resorted to, the body inverted, and several drops of nitrite of amyl placed at his nostrils. Recovery took place though it is evident that he had a narrow escape. In a large proportion of cases the action of bromide of ethyl is described as more free from unpleasant results than either chloroform or ether. In a few cases only have sickness or other bad results ensued, and when sickness has occurred it has passed off sooner than the sickness of ether.—*Pacific Med. and Surg. Jour.*

---

*Death from Bromide of Ethyl.*—Dr. R. J. Levis, of Philadelphia, the distinguished advocate of Bromide of Ethyl, recently lost a patient under this anæsthetic at the Jefferson Medical College Hospital, Philadelphia. The patient was about to be operated upon for stone in the bladder, but died as the first incision was being made. Dr. Levis was present during the administration of the anæsthetic, and no doubt exercised every known precaution. This case speaks a painful warning, and sounds the death knell to this over praised agent.

Perfect security against death from anæsthesia is not possible. We believe when prejudice has ceased and calm, judicial testimony is offered that chloroform will yet regain professional confidence, and receive recognition as the most reliable and satisfactory of all anæsthetics up to this time employed.—*Maryland Medical Journal.*

---

*American Dental Association.*—At the session of the American Dental Association, held in Boston, the report on Section 2, on dental education, urged that the Association should fix a standard of preliminary education to be required by dental colleges. The question whether dentistry was a specialty of medical science or not was considered. Dr. Brophy urged the necessity of dentists having a thorough medical education and a complete knowledge of dental surgery. The Association voted to hold its next annual meeting in New York City.

---

*Dental Instruments Transmitting Syphilis.*—A middle-aged lady called on us for the treatment of several ulcers on the tongue and palate, and for severe pains extending from the mouth down the neck to the body. She said that a dentist had plugged a tooth with an instrument containing blood, which had just been drawn from the mouth of a youth. The woman had syphilis, was treated for it, and recovered. It is unquestionable that the dental instrument had been the medium of innoculating the body with syphilitic virus. How many innocent persons, including even little children, may be victims of dental carelessness and filth.—*Journal of Materia Medica.*

---

*Local Anæsthesia with Bromide of Ethyl.*—M. Terrilon stated, before the Paris Surgical Society, that he had employed the bromide of ethyl about a dozen times in operations with the thermo-cautery. In a minute or two a white patch indicating cutaneous anæsthesia is produced, and on the pulverization being continued insensibility of the tissues is produced to the depth of two centimetres. The production of the white patch is not essential, as anæsthesia may exist when it is absent. The results have proved very satisfactory, but in two cases M. Terrillon did not succeed, owing, as he believes, to the pulverizators which he employed having too small a jet.—*Druggists Circular.*

---

*Changes in the Maxillary Bones in Ataxics.*—M. Vallin has observed, in several cases of locomotor ataxia, a falling out of the teeth which is not preceded by pain or caries. This phenomenon is due to a bony rarefaction of the alveolar border, as a result of which the teeth simply drop out of the alveoli. It is a trophic alteration of the maxillæ which has not hitherto been described, but which deserves careful study, as it may be an early symptom of the general disease. MM. Luys and Lereboullet have also observed this falling out of the teeth in ataxia. —*Le Courrier Medical.*

---

*Local Anæsthesia by Bromide of Ethyl.*—M. Perier, of Paris, states that he has employed the bromide of ethyl several times as a local anæsthetic with considerable success. It has the advantage over ether of not being inflammable, and hence can be employed when the actual cautery is to be used.—*La France Medicale.*

